# New Publications

**Published:** 1898-02

**Authors:** 


### NEW PUBLICATIONS.

Clinical Diagnosis of Lameness in the Horse. By W. E. A. Wyman, V.S., Professor of Veterinary Science at Clemson Agricultural and Medical College, South Carolina. Published by Wm. R. Jenkins, New York City.

This work, of about 175 pages, is a very carefully prepared exposition of the always interesting and important subject of lameness from a clinical standpoint. While it is prepared in such form as to be readily understood by the student, arranged in chapters, with a copious index, glossary, and a number of illustrations, it will be equally worthy of perusal by the practitioner. All forms of lameness, the structures involved, and local and constitutional causes and symptoms, general and special, are well elucidated, and the table of contents and index make it possible to quickly review any obscure case one might have, and find much to aid one in making a diagnosis. The many points of importance of this subject will find a welcome refreshing in the minds of the everyday practitioner, and some additional experiences by well-known practitioners have been added which are of much interest. The author acknowledges his indebtedness to Prof. Möller, and has quoted other eminent authorities on lameness, and presents a book worthy of recognition by our colleges and wherever veterinary science is taught. The typographical appearance and excellent paper add much to its attractiveness, at the same time adopting a popular form and size. The author has dedicated this contribution to his alma mater, the New York College of Veterinary Surgeons and School of Comparative Medicine, a grateful recognition of the earnest work being done by this institution as a school of instruction.

The University of Pennsylvania Catalogue is out for the year 1897–98. It is a book of nearly five hundred well-filled pages, containing a short account of every department, its instructors, facilities offered, expenses, list of students, and a budget of information relative to the work done by this well-known institution. A complete list of the graduates of every department in 1897, and a list of the various societies identified with the college, with an extended index, complete the catalogue.

---

A Viceregal Visit. A few minutes after noon, December 22d, Lord Aberdeen visited the Ontario Veterinary College, Toronto, where he was received by the Principal, Professor Smith, and the members of the Faculty. His Excellency was conducted through the museum, lecture-rooms, and laboratories of the college, and expressed himself as much pleased at the excellence of the arrangements, especially noticing with pleasure the admirable specimens in the museum. Reaching the large and beautifully-arranged histological rooms, the party found the students asembled, who received the Governor-General with applause. Principal Smith, in his capacity as chairman, alluded in graceful terms to the kindness of the Governor-General in visiting the college, and requested from his Excellency a few words. Lord Aberdeen graciously responded, and, in the course of his address, referred in felicitous terms to the presence of so many American students at this college and to the great reputation enjoyed by the institution in all parts of the English-speaking world. At the conclusion of his remarks a hearty vote of thanks to the Governor-General for his visit was proposed by Dr. Thorburn, seconded by Dr. Duncan, and carried with great applause. The proceedings terminated with three hearty cheers for the Governor General, after which he inspected some of the fine saddle and other horses in the stables of the college.

